# Canadian Women in Otolaryngology—Head and Neck Surgery part 1: the relationship of gender identity to career trajectory and experiences of harassment

**DOI:** 10.1186/s40463-023-00629-6

**Published:** 2023-04-24

**Authors:** Khrystyna Ioanidis, Kendra Naismith, Agnieszka Dzioba, S. Danielle MacNeil, Josée Paradis, Smriti Nayan, Julie E. Strychowsky, M. Elise Graham

**Affiliations:** 1 Schulich School of Medicine and Dentistry, Western University, London, Canada; 2 Department of Otolaryngology – Head and Neck Surgery, Western University and London Health Sciences Centre, London, ON Canada; 3 Division of Otolaryngology - Head and Neck Surgery, Cambridge Memorial Hospital, McMaster University, Hamilton, ON Canada

**Keywords:** Gender inequity, Women in surgery, Otolaryngology, Harassment, Career advancement

## Abstract

**Introduction:**

Women in surgical specialties face different challenges than their male peers. However, there is a paucity of literature exploring these challenges and their effects on a Canadian surgeon’s career.

**Methods:**

A REDCap® survey was distributed to Canadian Otolaryngology–Head and Neck Surgery (OHNS) staff and residents in March 2021 using the national society listserv and social media. Questions examined practice patterns, leadership positions, advancement, and experiences of harassment. Gender differences in survey responses were explored.

**Results:**

183 completed surveys were obtained, representing 21.8% of the Canadian society membership [838 members with 205 (24.4%) women]. 83 respondents self-identified as female (40% response rate) and 100 as male (16% response rate). Female respondents reported significantly fewer residency peers and colleagues identifying as their gender (p < .001). Female respondents were significantly less likely to agree with the statement “My department had the same expectations of residents regardless of gender” (p < .001). Similar results were observed in questions about fair evaluation, equal treatment, and leadership opportunities (all p < .001). Male respondents held the majority of department chair (p = .028), site chief (p = .011), and division chief positions (p = .005). Women reported experiencing significantly more verbal sexual harassment during residency (p < .001), and more verbal non-sexual harassment as staff (p = .03) than their male colleagues. In both female residents and staff, this was more likely to originate from patients or family members (p < .03).

**Discussion:**

There is a gender difference in the experience and treatment of OHNS residents and staff. By shedding light on this topic, as a specialty we can and must move towards greater diversity and equality.

**Graphical Abstract:**

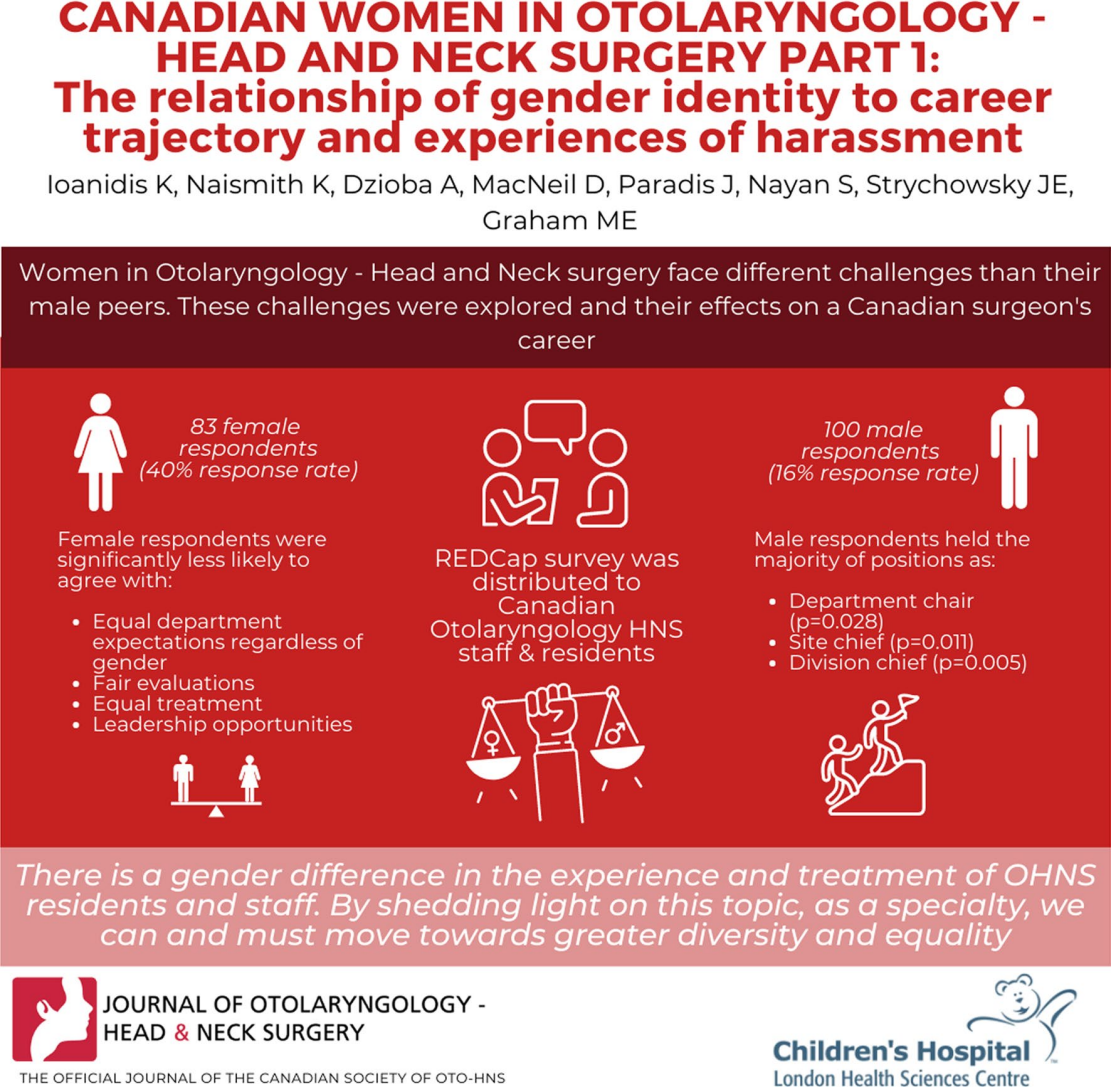

**Supplementary Information:**

The online version contains supplementary material available at 10.1186/s40463-023-00629-6.

## Background

There is increasing awareness in the literature and in popular media about gender inequity in medicine. Though women in medicine are increasing in numbers, there are many barriers that still exist. As of 2017, 40% of practicing physicians and 63% of medical students in Canada were women, and a recent Canadian Medical Association (CMA) study projected that by 2030 over 50% of physicians will identify as female [1]. However, there is yet to be a proportional rise in women in leadership roles. A recent 2020 study showed that only 18.6% of residency and fellowship directors and 5.1% of department chairs in the United States are women, and that significantly less of those female directors had achieved full professor rank as opposed to their male counterparts [2]. Monetary compensation has also been shown to be statistically different between male and female physicians. Even accounting for location and years in practice, female physicians in Ontario bill approximately 74% of what their male counterparts do [3, 4]. While some might attribute this to differences in hours worked, this myth has been dispelled [4].

Though identifying inequities is an important first step, it is much more complex to examine what factors perpetuate this inequity. Factors that have been previously attributed to the attrition of female physicians in academic medicine include lack of mentorship and role models, professional isolation, work-life imbalance, salary inequities, bullying and harassment [5]. In this two-part investigation, Canadian otolaryngologists were surveyed on the influence of gender on career progression and harassment in the workplace (Part I) and family, fertility, and lactation (Part II). The present paper reports on gender differences in career progression and harassment.

## Methods

### Data collection

Institutional ethics board approval was obtained from Western University (REB# 118,283). This study was a survey of Canadian OHNS attendings (both in active practice and retired) and trainees in accredited OHNS training programs. Physicians from specialties other than OHNS were excluded. The survey was available from March to May 2021. The survey was sent via the national listserv to 838 potential respondents (551 consultants, 118 emeritus status, and 169 residents). This was then promoted via social media including Facebook, Instagram, and Twitter. Topics addressed included demographics, residency experience, leadership and advancement, and harassment (Additional file 1). Survey development involved a literature search identifying previous relevant studies for reference and question adaptation, followed by an iterative process of survey generation and editing by all authors, representing academic and community otolaryngologists in several subspecialties as well as a resident in training to capture a breadth of experience. It was then translated into French to allow participation from all Canadian otolaryngologists. Responses were collected in REDCap® (Version 11.1.13. Copyright © 2020 REDCap) and compared based on respondent gender. Survey responses were anonymous.

### Data analysis

Completed surveys were excluded if they did not include demographic data or if they exclusively included demographic data. Descriptive statistics including means, standard deviations and frequencies of study outcomes were evaluated. Gender and career differences in study outcomes were explored with chi-squared tests for categorical outcomes and independent sample t-tests for continuous variables. We employed an alpha level of 0.05 to determine statistical significance. Data was processed and analyzed using R [6].

## Results

### Demographics

At survey closure, a total of 183 surveys were returned representing 21.8% of the potential respondents. Of those, 100 respondents identified as male (of 633 potential responses—a 16% response rate) and 83 identified as female (of 205 potential responses—a 40% response rate). Of the respondents, 120 (66%) identified as attending physicians and 62 (34%) identified as resident physicians. One respondent did not specify. The surveys represented a variety of subspecialties, practice environments and years of practice experience. Of practicing physicians that specified, 54 (45%) were in academic practice, 35 (29%) practiced in the community, and the remaining 30 (25%) were in a hybrid community-academic practice. There was no significant difference between genders in practice setting (χ^2^ = 0.59, p = 0.75) or resident intent to subspecialize (χ^2^ = 7.57, p = 0.37), however there was a significant difference in gender of attending surgeon by practice subspecialty (χ^2^ = 22.8, p < 0.001). Male respondents were more likely to have subspecialized in head and neck surgery (38%) while female respondents were more likely to have subspecialized in pediatric otolaryngology (35%). In general, female respondents were less likely to report having colleagues of the same gender, either as residents or as staff (p < 0.001).

### Gender-based inequality

Participants were asked to what degree they agreed with statements regarding equality of treatment of residents and attendings based on gender, rated on a Likert scale. Seventy-four percent of male respondents agreed while only 39% of female respondents agreed that their department had the same expectations of residents regardless of gender. Sixty-nine percent of male respondents agreed while only 35% of female respondents agreed that residents of all genders were evaluated fairly based on the same criteria. Sixty-five percent of male respondents agreed while only 36% of female respondents agreed that their program treated all residents equally regardless of gender. Finally, 65% of male respondents agreed while only 36% of female respondents agreed that the same leadership opportunities were open to everyone regardless of gender. In each case, the female respondents were significantly less likely to agree to the statement (all p-values < 0.01).

### Leadership

The survey included questions about academic rank, leadership positions and time taken to achieve these academic positions. While there was no statistical difference in academic rank between genders (χ^2^ = 9.99, p = 0.07), it is worth noting that only one female respondent of the 46 respondents to this question had achieved the rank of full professor (2.2%) vs twelve male respondents (12/72, 16.7%). There was a significant difference between male and female respondents identified in many leadership positions, however. There were 64 male and 38 female respondents to this question. Twelve male respondents (19%) identified as a department chair or chief as opposed to one female respondent (2.6%, p = 0.028). There were also 21 male site chiefs (33%) as opposed to 4 female (11%) (p = 0.011), and 18 male division chiefs (28%) as opposed to 2 female (5.3%, p < 0.01). A significantly higher proportion of female respondents reported having no leadership roles (17/38, 45%) as compared to their male colleagues (14/64, 22%) (p = 0.015). There was no significant difference between genders in the role of program director, assistant program director or rotation supervisor (Table 1).

**Table 1:** Leadership roles in male and female otolaryngologists

Leadership role	Male, N = 64	Female, N = 38	p value
Department chair/chief	12 (19%)	1 (2.6%)	**.028**
Site chief	21 (33%)	4 (11%)	**.011**
Division chief	18 (28%)	2 (5.3%)	** < .01**
Program director	12 (19%)	2 (5.3%)	.056
Assistant program director	1 (1.6%)	2 (5.3%)	.60
Rotation supervisor	27 (42%)	13 (34%)	.4
Other	8 (12%)	4 (11%)	> .9
None	14 (22%)	17 (45%)	**.015**

Bolded values indicate statistical significance

### Harassment

**Fig. 1:**
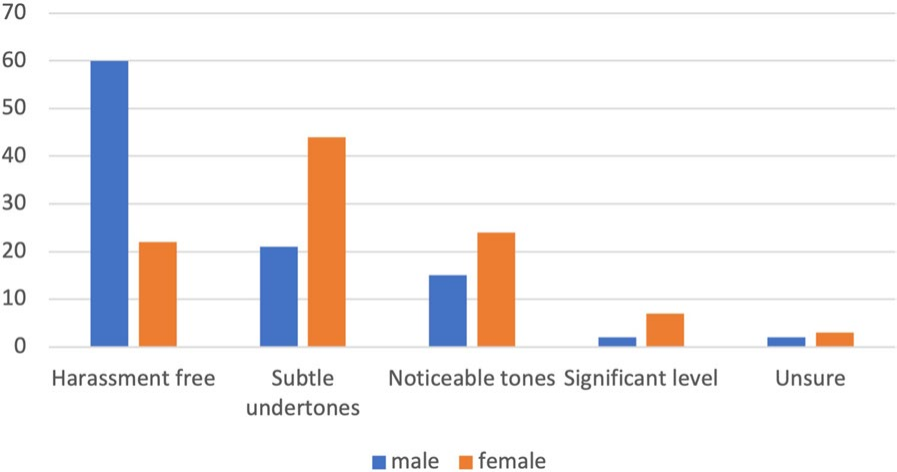
Perceived levels of harassment during residency. Female residents and staff reflecting on their residency were significantly more likely to report higher levels of harassment (p < .001)

Respondents were asked whether they had experienced harassment during their residency training and whether they would quantify the amount as “harassment free”, “subtle undertones”, “noticeable tones”, “significant” or “unsure”. Female respondents were significantly more likely to report higher levels of harassment (p < 0.01) than their male counterparts (Fig. 1). Respondents experiencing harassment were then asked to qualify the type of harassment as verbal (non-sexual), sexual (verbal), sexual (physical), racial/ethnic, or physical (Table 2). The only type of harassment with a statistical difference between male and female respondents was sexual (verbal) harassment (p < 0.001). When asked who was responsible for this harassment, male respondents reported statistically more harassment in residency by leaders in their department (p = 0.02), where female residents experienced more harassment from patients or patient family members (p < 0.01). There was no statistical difference between genders for harassment from colleagues/other residents, ancillary staff or administration (Table 3). Overall, the incidence of harassment was high during training, with 37.9% of male and 75.2% of female respondents experiencing some harassment during training. Of those reporting harassment, 87% of female and 92% of male respondents reported this harassment was verbal non-sexual. Forty-five percent of female respondents reported experiencing verbal sexual harassment during their training while only 11% of male residents reported the same.

**Table 2:** Types of harassment experienced by respondents

Harassment type during residency training	Male, N = 37	Female, N = 53	p value
Verbal (non-sexual)	34 (92%)	46 (87%)	.5
Sexual harassment (verbal)	4 (11%)	24 (45%)	** < .01**
Sexual harassment (physical)	0 (0%)	2 (3.8%)	.5
Racial/ethnic harassment	9 (24%)	8 (15%)	.3
Physical harassment (non-sexual)	1 (2.7%)	5 (9.4%)	.4

Harassment type—attending	Male, N = 23	Female, N = 27	p value
Verbal (non-sexual)	15 (65%)	25 (93%)	**.03**
Sexual harassment (verbal)	5 (22%)	11 (41%)	.2
Sexual harassment (physical)	1 (4.3%)	1 (3.7%)	> .9
Racial/ethnic harassment	10 (43%)	2 (7.4%)	** < .01**
Physical harassment (non-sexual)	2 (8.7%)	0 (0%)	.2

Bolded values indicate statistical significance

Those respondents reporting having experienced harassment were asked to describe the nature of this harassment, and to choose all that apply. “Residents” includes both those currently in training and attending physicians reflecting on their training

**Table 3:** Perpetrators of harassment

Experienced by residents	Male, N = 37	Female, N = 53	p value
Leaders in my department	27 (73%)	25 (47%)	**.02**
Colleagues/other resident	14 (38%)	25 (47%)	.4
Patients or family members	10 (27%)	29 (55%)	** < .01**
Ancillary staff	14 (38%)	20 (38%)	> .9
Administration	5 (14%)	8 (15%)	.8

Experienced by attendings	Male, N = 23	Female, N = 24	p value
Leaders in my department	11 (48%)	7 (29%)	.2
Colleagues	13 (57%)	15 (62%)	.7
Residents	1 (4.3%)	4 (17%)	.3
Patients or family members	8 (35%)	16 (67%)	**.03**
Ancillary staff	3 (13%)	7 (29%)	.3
Administration	5 (22%)	2 (8.3%)	.2

Bolded values indicate statistical significance

Those respondents reporting having experienced harassment were asked to describe who was responsible for the harassment, and to choose all options that apply. “Residents” includes both those currently in training and attending physicians reflecting on their training

**Fig. 2:**
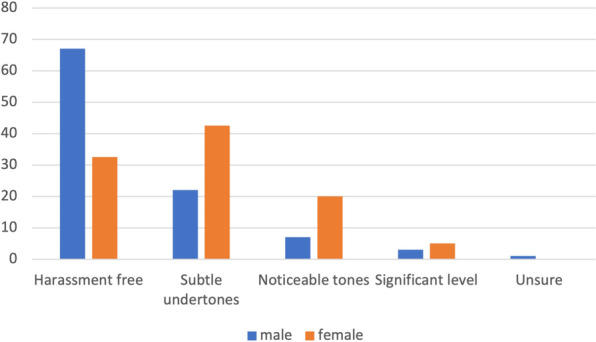
Perceived levels of harassment among attending staff. Female attending surgeons were significantly more likely to report harassment than their male colleagues (p = .003)

Similar responses were endorsed by attending physicians. Overall, 31.6% of male and 68% of female attending staff reported some level of harassment, with the degree of harassment experienced by female staff being higher (p < 0.01, Fig. 2). When asked to categorize the type of harassment, female attending staff were more likely to have experienced verbal nonsexual harassment (p = 0.03), while male attending staff were more likely to have experienced racial or ethnic harassment (p < 0.01) (Table 2). When asked who was responsible for this harassment, female respondents reported significantly more harassment from patients and family members (p = 0.03) (Table 3). Of those experiencing harassment, 93% of female attending respondents and 65% of male attending respondents reported verbal non-sexual harassment. Forty-one percent of female attending physicians who had experienced harassment reported verbal sexual harassment while only 22% of male attending staff reported the same. This difference was not statistically significant (p = 0.2).

A free text response was included as part of the section of the survey on harassment. The responses were varied and poignant. There were 32 total responses provided within this section. Thematic analysis identified prominent themes of reporting experiencing harassment, most commonly verbal, and most often from colleagues. One respondent said: “Verbal harassment on an everyday basis which are mentally draining and annoying”. Another respondent said: “As a female junior resident, navigating relationships with nursing staff (OR, ICU, ward) or administrative staff can be challenging. Differential treatment to female vs. male trainees can be obvious, and was sometimes even hostile to women. Some hospital sites, especially those with fewer/infrequent trainees, were noticeably worse for this”.

## Discussion

This large survey of Canadian otolaryngologists serves to highlight the significant differences in the experiences of male and female residents and practicing physicians in this specialty.

In particular, there are significant differences in leadership roles attained by female physicians as compared to male colleagues. While there was no statistical difference in the academic rank of the respondents within our survey, there was a significant lack of female representation in high level leadership positions such as program director and department chair. The reasons for these discrepancies are complex and likely interrelated.

In terms of inequality between genders, female respondents were less likely to agree that there was equal treatment between genders starting as early as residency training. While it is impossible to quantify “equal treatment”, the fact that this perceived discrepancy exists so early on may discourage female otolaryngology residents from aspiring to an academic career or a position in leadership. In addition, 43% of surveyed American female otolaryngologists reported that having a child had influenced their decision regarding department or practice leadership roles [7]. Having role models in leadership positions encourages young surgeons and current or future parents to see themselves in those positions in the future. As such, increasing female representation now is integral to ensuring gender diversity in leadership in the future.

Harassment can also be a barrier to progression in academic rank and leadership and is not unique to any particular specialty in medicine. Recent surveys of female French intensivists and female American anesthesiologists report high levels of harassment at work [8, 9]. A recent survey of the Women in Otolaryngology section of the American Academy of Otolaryngology noted that 41% of respondents had experienced at least a subtle level of harassment during their residency training [7]. In our study we were able to show that women report a higher level of harassment both in residency and as an attending physician. Female respondents also endorsed more harassment from patients and colleagues than their male peers. This correlated with a lower rate of female identified surgeons in leadership positions. There is a significant amount of literature that shows that women are less likely to pursue an academic medical or surgical career [5, 10]. While this study was not powered to link levels of harassment with academic progression, one can surmise that this could be one of many reasons that female surgeons are less likely to hold leadership positions in their academic institutions.

Harassment not only limits academic advancement. Workplace violence, including verbal harassment, has been linked to increased objective measures of burnout, decreased job satisfaction, and attrition [11–13]. Workplace violence among healthcare workers may also cause psychological distress, decreased sleep and ultimately has downstream effects on the quality of patient care [14]. It is clear that minimizing harassment for all members of the care team is a crucial step in improving the experience of physicians and patients alike.

Once we can ascertain that women in otolaryngology are less likely to hold leadership positions and more likely to experience harassment, we then can start to discuss ways to mitigate these issues. In medicine, we can learn from other sectors that have already started to bridge this gap. For example, in 2018 the Harvard Business Review discussed ways to encourage women on a path to leadership in medicine [15]. Since women often have other significant roles in their families such as childcare or caring for ill family members, they highlighted that a work-family balance would be integral to supporting that goal. Progressive policies for family leave, support for lactating parents, and childcare policies such as on-site or emergency back-up childcare could support women in furthering their academic and leadership careers. Career flexibility such as the option to telecommute, flex-time, and flexibility in academic promotion also could encourage more women to include leadership roles or academic duties in their career progression. In terms of mitigating bias and harassment in the workplace they recommended implicit bias training for all staff and leaders, salary reviews to hold departments accountable for overt discrepancies between genders, and better reporting systems and legal support for those who have experienced harassment. Finally, they suggested that both formal and informal mentorship and sponsorship between women would be integral to including more female physicians at the highest levels of leadership. While implementing all those suggestions would be a fundamental shift in the way careers in medicine are currently structured, it would not only encourage gender diversity at the highest levels of medical leadership but would improve work-life balance for our male colleagues as well.

The proportion of female respondents to the survey was significantly higher than male, with 40% and 16% response rates, respectively, and this introduces the potential for selection bias. There may be several reasons for this observed difference in response rate. The survey title hinted at its contents, which included “Fertility, family planning, and lactation,” which may have resonated more with female otolaryngologists. Harassment was also mentioned in the title, perhaps leading to an increased likelihood of responses from those who had experienced harassment, both male and female. It is possible that the experience of male otolaryngologists in particular was not completely captured in the population that chose to participate. However, the high response rate of women otolaryngologist also represents a strength of this study. With over 40% of the Canadian Society of Otolaryngology’s female membership represented, the data likely relatively accurately depicts the current landscape for female otolaryngologists in Canada. An additional strength is in the building of the survey tool: despite not being validated, it was edited and vetted by multiple members of the otolaryngology community and underwent translation to allow participation from our French Canadian otolaryngology colleagues.

The study has several limitations, including overall low response rate (21.8%) and the use of the Canadian Society of Otolaryngology Listserv for distribution, as not all Canadian otolaryngologists are members of this society. In addition, the focus on the Canadian population may mean it is less generalizable to other populations, though several studies in other countries and specialties suggest our findings are similar [2, 5, 7, 9, 14, 16–18]. The survey utilized was not validated. Finally, all of the respondents identified as male or female, so we do not have data on career advancement or harassment in non-binary identified individuals. Ideally a higher response rate would improve (but not guarantee) the chance of exploring the full diversity of experience based on the spectrum that is gender identity.

## Conclusions

Based on this large-scale survey of Canadian otolaryngologists, we have been able to demonstrate that female otolaryngologists occupy fewer leadership roles and experience higher levels of harassment than their male peers both in training and afterwards. Female otolaryngology trainees also perceive a lower level of gender equality during their training as opposed to their male peers. By identifying these trends, we can work towards a safer and more equitable workplace.

## Supplementary Information

**Additional file 1**. Survey material distributed to Canadian Society of Otolaryngology—Head and Neck Surgery membership.

## Data Availability

The complete survey is made available in Additional file 1. Survey data can be made available upon request.
