# Supplementary material for: Canadian Women in Otolaryngology—Head and Neck Surgery part 1: the relationship of gender identity to career trajectory and experiences of harassment
Source: J Otolaryngol Head Neck Surg. 2023 Apr 24;52:31. doi: 10.1186/s40463-023-00629-6 (PMC10127062; doi:10.1186/s40463-023-00629-6)
Supplement: Supplementary file 1 — Additional file 1. Survey material distributed to Canadian Society of Otolaryngology—Head and Neck Surgery membership. [file 40463_2023_629_MOESM1_ESM.pdf]

Survey: Influence of gender identify on family planning and professional advancement in otolaryngology-head and neck surgery

Demographics

What is your age (in years)? __________________________________

What is your gender identity?

Agender/genderqueer or genderfluid/man/non-binary/questioning or unsure/two-spirit/woman/prefer not to disclose/additional gender category ______

What is your sexual orientation?

Aromantic/Asexual/Bisexual/Fluid/Gay/Lesbian/Pansexual/Queer/Questioning or unsure/Same gender-loving/Straight (heterosexual)/Prefer not to disclose/Additional category/identity no listed (please specify) ______

What pronouns do you use? (select all that apply)

[he/him]/[she/her]/[they/their]/other, please specify ______

What is your race/ethnicity? (select all that apply)

Asian - East (eg, Chinese, Japanese, Korean)/Asian - South (eg, Indian, Pakistani, Sri Lankan)/Asian - Southeast (eg, Malaysian, Filipino, Vietnamese)/Black - African (eg, Ghanaian, Kenyan, Somali)/Black - Caribbean (eg, Barbadian, Jamaican)/Black - North American (eg, Canadian, American)/First Nations Indian - Caribbean (eg, Guyanese with origins in India)/Indigenous or Aboriginal/not included elsewhere/Inuit Latin American (eg, Argentinian, Chilean, Salvadoran)/Metis/Middle Eastern (eg, Egyptian, Iranian, Lebanese)/White - European (eg, English, Italian, Portuguese, Russian)/White - North American (eg, American, Canadian)/Mixed heritage (eg, black African and white North American)/Other please specify ______/Prefer not to answer/Do not know

What is your practice setting?

Academic/Community/Community-Academic

What is your marital status?

Married or Common-Law/Single/Cohabiting/Divorced or Separated/Widowed/Prefer not to specify/Other, please specify ______

What province are you located in?

Alberta/BC/Manitoba/New Brunswick/Newfoundland/Nova Scotia/Ontario/PEI/Quebec /Saskatchewan/NWT/Nunavut/Yukon

What is your stage of practice?

Resident or Fellow/Attending staff/Retired

[If Fellow] What PGY year are you in?

1/2/3/4/5

[If Fellow] Do you intend to subspecialize?

No or General/Facial Plastics/Head and Neck Surgery Laryngology/Otology/Pediatric Otolaryngology/Rhinology/Undecided

[If Attending] How many years have you been in practice?

Less than 5/5 – 9/10 – 14/15 – 19/20 or more

[If Attending] On average, how many days do you work (clinical and non-clinical) per week?

5 or more/3 – 4/Less than 3

[If Attending] Did you complete additional fellowship/subspecialty training?

Yes/No

[If Attending] What is your primary subspecialty?

General/Facial Plastics/Head and Neck Surgery/Laryngology/Otology/Pediatric Otolaryngology/ Rhinology

[If Attending] What is your secondary specialty? (select all that apply)

General/Facial Plastics/Head and Neck Surgery/Laryngology/Otology/Pediatric Otolaryngology/ Rhinology/None

[If Attending] What is your current academic rank?

Lecturer/Assistant Professor/Associate Professor/Full Professor/Other, please specify ______ /None

[If Attending] How many years did it take to obtain this ranking? (enter number of years) _________

Residency Training

What was the percentage of same gender co-residents over the duration of your residency?

0-20%/20-40%/40-60%/60-80%/80-100%

What was the percentage of same gender attending staff while in residency?

0-20%/20-40%/40-60%/60-80%/80-100%

Please state your agreement with the following statements about department/division leaders during residency: disagree/somewhat disagree/neutral/somewhat/agree/agree/N/A

a) My department leaders were supportive of residents starting families
b) My department was supportive of time away for maternity leave
c) My department was supportive of time away for paternity leave
d) My department allowed me to take time away for family issues
e) My department had the same expectations of residents regardless of gender

f) Residents of all genders were evaluated fairly based on the same criteria
g) My program leaders treated all residents equally regardless of gender
h) The same leadership opportunities were open to everyone regardless of gender

Please state your agreement with the following statements about your co-residents during residency: disagree/somewhat disagree/neutral/somewhat agree/agree

a) My female co-residents were supportive of women starting families
b) My male co-residents were supportive of women starting families
c) My female co-residents were supportive of those taking time away for family issues
d) My male co-residents were supportive of those taking time away for family issues

How would you rate your residency experience overall?

Excellent/very good/good/fair/poor

What would you change about your residency program? ____

Leadership

[If Attending] What are your leadership roles (past and present)? (select all that apply)

Department Chair or Chief/Site Chief/Division Chief/Program Director/Assistant Program Director/Rotation Supervisor/Other ______/None

In your current department, what percentage of the following roles are held by women? [response options: 0-20%/20-40%/40-60%/60-80%/80-100%]

a) Physicians
b) Department Chair/Chief
c) Site Chief
d) Division Chief
e) Program Director
f) Assistant Program Director
g) Rotation Supervisor

Harrassment

Harassment is a form of discrimination. It includes any unwanted physical or verbal behaviour that offends or humiliates you. Generally, harassment is a behaviour that persists over time. Serious one-time incidents can also sometimes be considered harassment. Harassment occurs when someone: makes unwelcome remarks or jokes about your race, religion, sex, age, disability or any other of the grounds of discrimination; threatens or intimidates you because of your race, religion, sex, age, disability or any other of the grounds of discrimination; makes unwelcome physical contact with you, such as touching, patting, or pinching. Canadian Human Rights Commission (https://www.chrc-ccdp.gc.ca/eng/content/what-harassment-1)

Did you experience harassment during residency?

Harassment free/Subtle undertones of harassment/Noticeable tones of harassment/Significant level of harassment/Unsure

[If yes to harassment question] Types of harassment experienced during residency (Select all that apply):
Verbal (non-sexual)/Sexual harassment (verbal)/Sexual harassment (physical)/Racial or Ethnic harassment/Physical harassment (non-sexual)

[If yes to harassment question] Who was responsible for the harassment? (select all that apply)
Leaders in my department/Colleagues or Other resident/Patients or family members/Ancillary Staff/Administration

[If Attending] Did you experience harassment at work?
Harassment free/Subtle undertones of harassment/Noticeable tones of harassment/Significant level of harassment/Not sure

[If yes to harassment question] Types of harassment experienced post-residency/in practice (select all that apply)
Verbal (non-sexual)/Sexual harassment (verbal)/Sexual harassment (physical)/Racial or Ethnic harassment/Physical harassment (non-sexual)

[If yes to harassment question] Who was responsible for the harassment? (select all that apply)
Leaders in my department/Colleagues or Residents/Patients or family members/Ancillary Staff/ Administration

Please provide additional comments on your experience with harassment: ________________________

Work-Life Balance
Please state your agreement with the following statements: [response options: disagree/somewhat disagree/neutral somewhat agree/agree]

a) I feel comfortable as a [gender_identity] at work
b) It is easy managing work-life balance
c) I am happy with my decision to be an otolaryngologist-head and neck surgeon
d) I would recommend otolaryngology-head and neck surgery as a career to a young [gender_identity]

Children, Lactation, and Parental Leave
Do you have children?
Yes/no/prefer not to answer

[If no to children] Have you ever been pregnant?
Yes/No

[If no to children] Do you plan to have children in the future?
Yes/no/unsure

[If yes to children or yes to ever pregnant] Did you/your partner experience any miscarriages?

Yes, how many? ______/No

[If yes to children or yes to ever pregnant] Did you/your partner experience any stillbirths

Yes, how many? ______/No

[If yes to children or yes to ever pregnant] Did you/your partner have any therapeutic abortions?

Yes, how many? ______/No

[If yes to children or yes to ever pregnant] Did you/your partner have any abnormal prenatal screening during any pregnancy?

Yes/No

[If yes to abnormal prenatal screening] Please describe further your experience with miscarriage and pregnancy complication: ____

Please state your level of agreement with the following statements: [response options: disagree/ somewhat disagree/neutral/somewhat agree/agree]

a) Training/practice influenced my decision to have children
b) Training/practice influenced my decision about WHEN to have children
c) Training/practice influenced my ABILITY to have children
d) I have concerns about future family planning
e) I have concerns about future fertility
f) I have concerns about future maternity/paternity leave

Have you accessed fertility services?

Yes/No

Have you used assistive reproductive technologies/IVF?

Yes/No

If you have experienced difficulty with conception, what was your underlying diagnosis (if given)? ___________________________________

Please provide additional comments regarding fertility and family planning: ________________________

[If yes to children] How many children do you have? ___________________________________

[If yes to children] How old were you when your first child was born? ____________________________

[If yes to children] When were your children born? (Check all that apply)

Before medical school/Medical school/Residency or Fellowship/Clinical Practice/Other

[If yes to children] Did you take maternity/paternity leave during medical school, residency or clinical practice?

Yes/No

[If yes to mat/pat leave during med school/residency/clinical practice] On average, how much time did you take for maternity/paternity leave during medical school/residency/clinical practice (please enter number of weeks)? __________________

[If yes to mat/pat leave during med school/residency/clinical practice] Did you take as much leave as you planned/intended during medical school/residency/clinical practice?

Yes/No

If you have a partner, did your partner take leave?

Yes/no/not applicable

[If yes to partner taking leave] On average, how long did your partner take leave? (please enter number of weeks) ________________

[If yes to maternity/paternity leave] Please state the extent to which the following factors influenced your length of leave: [response options: Did not affect/Minor affect/Neutral/Moderate affect/Strong affect/N/A]

a) Support of partner/family/friends
b) Concern about losing skills
c) Concern about future opportunities
d) Concern about stigma
e) Pressure from supervisors
f) Pressure from colleagues
g) Financial concerns
h) Difficulty finding coverage for practice
i) Difficulty finding child care
j) Miss working/seeing patients
k) Other, please specify ______

Training/practice influenced the number of children I CHOSE to have?

Disagree/somewhat disagree/neutral/somewhat agree/agree

Training/practice influenced my ABILITY to conceive as many children as I wished to have?

Disagree/somewhat disagree/neutral/somewhat agree/agree

Did you access fertility services to help conceive a child?

Yes/No

Did you use assistive reproductive technologies?

Yes/No

Did you choose adoption?

Yes/No

If you had difficulty with conception, what was your underlying diagnosis? ________________________

Maternity/Paternity Leave and Advancement [If yes to children]

On average, at how many weeks gestational age did you stop call? _______________

Please indicate your agreement with the following statements: [response choices: disagree/somewhat disagree/neutral/somewhat agree/agree/N/A]

a) Colleagues were supportive of my pregnancy
b) Colleagues were supportive of my maternity/paternity leave
c) Having a child influenced my decision to pursue fellowship or additional post-residency education
d) Having a child influenced my decision to pursue department or practice leadership roles
e) Maternity/paternity leave impacted my number of opportunities for career advancement
f) Maternity/paternity leave impacted my salary/remuneration
g) Having a family changed my work hours/practice pattern

Please provide additional comments on how maternity/paternity leave has impacted advancement at work: ____

Lactation [If yes to children]

Did you CHOOSE and/or PLAN to breastfeed any of your children?

Yes/No

Which of your children did you breastfeed? (check all that apply)

1st child/2nd child/3rd child/4th child/5th child/6th child

Did you pump breastmilk at work as a resident?

No/Yes, for which child ______

Did you pump breastmilk at work as an attending staff?

No/Yes, for which child ______

How long did you pump at work (only the time while back at work, not in preparation for return)? [response options: < 1 month/1-3 months/3-6 months/>6months/N/A]

a) 1st child
b) 2nd child
c) 3rd child
d) 4th child
e) 5th child
f) 6th child

What age was your first/second/third/fourth/fifth/sixth child when they stopped receiving direct breastfeeds (please enter in months)?__________________________________

What age was your first/second/third/fourth/fifth/sixth child when they stopped receiving pumped breastmilk (please enter age in month)? _________________________________

Please state your agreement with the following statements: [response options: disagree/somewhat disagree/neutral/somewhat agree/agree]

a) I had adequate time to pump at work I had adequate space to pump at work (clean, private, accessible)
b) I had adequate space for pumped breastmilk storage at work
c) I met my breastfeeding goals
d) I felt supported in my decision to pump breastmilk at work
e) I experienced criticism or discrimination as a result of my need to pump breastmilk at work

Did you experience a breastfeeding complication (ie. reduced supply, blocked duct, mastitis) as a result of not being able to pump as frequently as you needed?

Yes/No

In your own words, please describe your experience pumping breastmilk at work: __________________

Final Thoughts Please list any topics that you would like to be addressed in future: __________________

Additional comments: ____
